# Does patient's expectation benefit acupuncture treatment? A protocol for systematic review and meta-analysis

**DOI:** 10.1097/MD.0000000000024178

**Published:** 2021-01-08

**Authors:** Zuoqin Yang, Yan Li, Zihao Zou, Ying Zhao, Wei Zhang, Huiling Jiang, Yujun Hou, Ying Li, Qianhua Zheng

**Affiliations:** a Department of Acupuncture and Moxibustion, Chengdu Pidu District Hospital of Traditional Chinese Medicine/the 3rd Affiliated Hospital of Chengdu University of Traditional Chinese Medicine (West District), No. 169, 1st Section of Zhongxin Avenue; b Department of Central Transportation Center, West China Hospital, Sichuan University, No. 28, Dianxin South Road; c School of Acupuncture–Moxibustion and Tuina; d Graduate School, Chengdu University of Traditional Chinese Medicine, No. 37 Shi’er Qiao Road, Chengdu, China.

**Keywords:** acupuncture, patient expectation, protocol, systematic review, treatment response

## Abstract

**Background:**

Patients’ expectation to treatment response is one source of placebo effects. A number of randomized controlled trials (RCTs) reported that expectation benefits to acupuncture treatment, while some did not. Previous systematic reviews failed to draw a confirmative conclusion due to the methodological heterogeneity. It is necessary to conduct a new systematic review to find out whether expectation can influence acupuncture outcomes.

**Methods:**

We systematically search English and Chinese databases from their inception to 3rd October, 2020, including MEDLINE, EMBASE, Cochrane Central Register of Controlled Trials (CENTRAL), Chinese BioMedical Literature Database (CBM), Chinese National Knowledge Infrastructure (CNKI), and Chinese Science and Technology Periodical Database (VIP). RCTs that evaluated the relationship between expectation and treatment response following acupuncture for adults will be included. Study selection, data extraction, and risk of bias assessment will be conducted independently. Risk of bias will be assessed by the Cochrane risk of bias assessment tool. Data synthesis will be performed by Review Manager (RevMan) software if the data is suitable for synthesis.

**Results:**

This systematic review will provide evidence that whether patients’ expectation impacts on the therapeutic effects of acupuncture. This protocol will be performed and reported according to the Preferred Reporting Items from Systematic Reviews and Meta-analysis Protocols (PRISMA-P) statement. The findings of this review will be disseminated through peer-reviewed publications and conference presentations.

**Conclusion:**

This systematic review aims to assess whether a higher level of patient's expectation contributes to a better outcome after acupuncture treatment, and in which medical condition this contribution will be more significant.

**INPLASY registration number:**

INPLASY2020100020 on International Platform of Registered Systematic Review and Meta-analysis Protocols.

## 1 Introduction

As one part of complementary therapies, acupuncture is well known by its therapeutic effects and safety in clinical practice. However, previous studies showed that verum acupuncture failed to show significant differences between sham acupuncture in randomized controlled trials (RCTs). While comparing with no treatment or usual care, verum acupuncture has better effectiveness.^[1–4]^ It suggests that a sizeable placebo effects may contribute to treatment response of acupuncture.

Patient's expectation has influence on outcomes in clinical practice as 1 part of placebo effects.^[5]^ Some studies reported that patients with optimistic expectation achieved better outcomes after acupuncture treatment.^[6–8]^ Two systematic reviews published in 2012 and 2015, respectively, reported that there did appear to a significant relationship between patient expectation and treatment responses, but without a confirm conclusion.^[9,10]^ It may because there were different medical conditions, methodology of included studies, expectation measurements, statistical methods, and so on. In these systematic reviews, most included studies focused on acupuncture analgesia, and better outcomes were easily observed when participants reported more expectations.^[6,7,11]^ It was reported that psychological states had a close relationship with expectation level.^[12,13]^ Therefore, is a patient with psychological disorder more likely to benefit from acupuncture treatment because of higher level of expectation. Furthermore, different assessment tools and statistical methods were also contributors to heterogeneity. It is needed to conduct subgroup analyses for these, which may provide more understanding of expectation in acupuncture studies.

Several new RCTs studying on the relationship between patients expectation and acupuncture responses have been published in recent 5 years.^[14,15]^ It is worth to perform a new systematic review or possible meta-analysis to detect whether patients’ expectation will influence on acupuncture outcomes. We sincerely hope that we will provide more evidence to estimate the placebo effect of acupuncture by this review.

## 2 Methods

### 2.1 Objectives

(1) Does a higher level of patient's expectation benefit acupuncture treatment in RCTs?
(2) In what kind of medical condition, a positive relationship between patients’ expectation and outcome improvements after acupuncture treatment can be detected easily?
(3) Do different expectation measurements and statistical methods affect the patients’ response to acupuncture?

### 2.2 Study registration

This protocol has been registered on International Platform of Registered Systematic Review and Meta-analysis Protocols with registration number INPLASY2020100020 (https://inplasy.com/inplasy-2020-10-0020/). This protocol follows the Preferred Reporting Items for Systematic Reviews and Meta-Analyses Protocols (PRISMA-P) statement guidelines.^[16]^

### 2.3 Criteria for study eligibility

#### 2.3.1 Types of studies

We will include RCTs or secondary analysis of RCTs which studied on the relationship between patient's expectation and treatment responses following acupuncture. Randomized, allocation concealment, and blinding methods should be clearly described in studies. But the blinding is not necessary for acupuncture because of its characteristics. We will exclude studies that patients’ expectation or medical condition induced by patient–doctor communication and experimental methods, respectively. Animal mechanism studies, cases reports, non-RCT studies will be excluded. In order to guarantee the quality of studies, only sample size of more than 30 will be considered. RCTs with crossover designs will not be included because of the washout duration of acupuncture cannot be accurately evaluated and the expectation level may change after acupuncture treatment.

#### 2.3.2 Type of participants

Adult patients aged ≥18 years old with any medical or psychological condition will be included. There are no restrictions on gender, race, economic status, or education.

#### 2.3.3 Types of intervention

The treatment group should be acupuncture, which includes manual acupuncture, electroacupuncture, auricular acupuncture, scalp acupuncture, intradermal needle, and transcutaneous electrical nerve stimulation (TENS) without moxibustion or Chinese medicine. Because combined methods, such as acupuncture with moxibustion or Chinese medicine decoction, will make the true effectiveness of acupuncture difficult to evaluate. Therefore, we will exclude studies use combined therapy.

#### 2.3.4 Types of controls

Controls include the following types will be included:

(1) Placebo controls: Sham acupuncture (e.g., needling at no-acupoint), placebo drugs/device (e.g., Park Sham Placebo Acupuncture Device), sham interventions (e.g., sham laser), and so on.
(2) Positive medication: Participants are administrated positive medication which were recommended by guidelines.
(3) No acupuncture treatment, such as waiting list: Participants receive no acupuncture treatment, or receive general care or usual care (e.g., health education, exercise recommendation). We will exclude the studies which only applying Chinese medicine, or other methods that we cannot identify the effects as a control, such as cupping or tuina.

#### 2.3.5 Types of outcome measures

Because eligible RCTs in any medical condition will be included, there are no constraints on health-related outcomes. Acupuncture expectation assessment or relevant information collection can be any type:

(1) Questions such as “What do you expect from this acupuncture treatment that you will receive for this disease?,” “How much improvement do you expect after acupuncture treatment?,” or “How much will your symptoms alleviate after acupuncture treatment?”;
(2) Questionnaire such as Credibility and Expectancy Questionnaire (CEQ),^[17]^ Acupuncture Expectancy Scale (AES).^[18,19]^ Expectation levels measured by Visual Analogue Scale (VAS)/Numerical Rating Scale (NRS) for continuous variables, or Likert scale for categorical variables.

The expectation should be assessed before acupuncture treatment. RCTs only collected expectation information after first or last session of acupuncture treatment will not be included. Because expectation level will change due to variable factors, such as the doctor–patient communication^[20,21]^ or response to treatment.^[22]^

### 2.4 Search strategy

A systematic search will be conducted in the following electronic databases: MEDLINE, EMBASE, the Cochrane Central Register of Controlled Trials (CENTRAL), Chinese BioMedical Literature Database (CBM), Chinese National Knowledge Infrastructure (CNKI), and Chinese Science and Technology Periodical Database (VIP) from inception to October 1, 2020. Relevant studies will be identified by systematic search as well as check reference lists of previous systematic reviews. Because of language limitation and quality assurance, only peer-reviewed publications in English or Chinese will be screened. Full articles or abstract will be included. The following search terms will be combined for systematic search, and Chinese terms will be used in Chinese databases:

(1) “acupuncture,” “acupuncture therapy,” “auricular acupuncture,” “transcutaneous electrostimulation/TENS,” “scalp acupuncture,” “manual acupuncture,” “electro-acupuncture,” “laser acupuncture";
(2) “randomized/randomised controlled trial,” “controlled clinical trial”;
(3) “expectation,” “expectancy,” “expected efficacy/effect,” “placebo effect.”

The search strategy for MEDLINE is shown in Table 1. The search strategy will be modified to be suitable for other databases.

**Table 1:** Searching items for identifying articles in MEDLINE.

Number	Search terms
1	randomized controlled trial.pt.
2	controlled clinical trial.pt.
3	(randomized or randomised).ab.
4	placebo.ab.
5	randomly.ab.
6	trial.ab.
7	groups.ab.
8	1 or 2 or 3 or 4 or 5 or 6 or 7
9	expectation^∗^.ab,ti. {including related terms}
10	expectancy.ab,ti. {including related terms}
11	placebo.ab,ti. {including related terms}
12	specific effect^∗^.ab,ti. {including related terms}
13	non-specific effect^∗^.ab,ti. {including related terms}
14	9 or 10 or 11 or 12 or 13
15	acupuncture.sh, ti, ab. {including related terms}
16	ear acupuncture.sh, ti, ab. {including related terms}
17	electroacupuncture.ab,ti. {including related terms}
18	acupuncture point^∗^.ab,ti. {including related terms}
19	acupressure^∗^.ab,ti. {including related terms}
20	meridians^∗^.ab,ti. {including related terms}
21	15 or 16 or 17 or 18 or 19 or 20
22	8 and 14
23	21 and 22

### 2.5 Data collection and extraction

#### 2.5.1 Study selection

**Figure 1:**
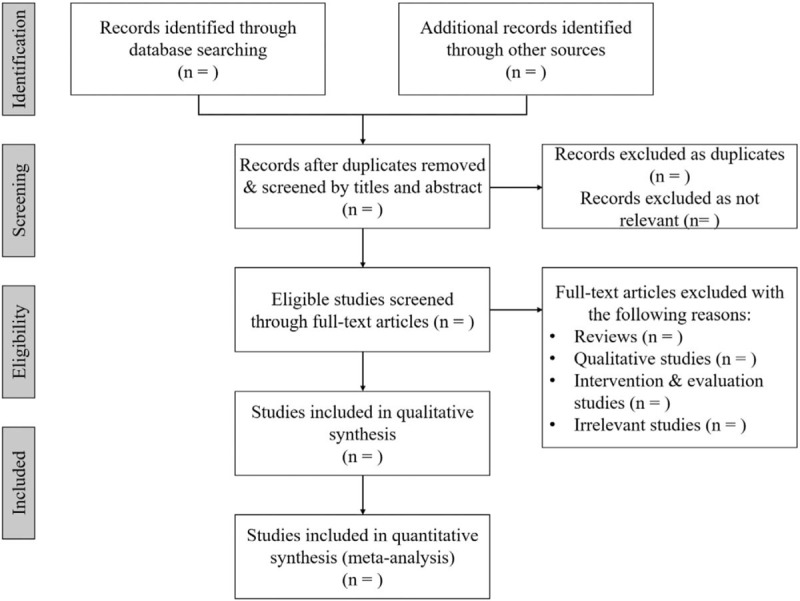
Flow chart for performing the systematic review.

We will import the details of retrieved articles from databases into EndNote V.X9 (Clarivate Analytics, Philadelphia, United States), and excluded irrelevant literature, and duplicated articles based on the titles and abstracts. Two reviewers (ZZH and JHL) will independently screen the titles and abstracts to exclude RCTs fail to meet eligible criteria. Full text of studies will be needed if the eligibility cannot be identified by titles or abstracts. Incomplete information will be obtained by contacting the authors. The results will be cross-checked by 2 reviewers (ZZH and JHL). Any disagreement will be resolved by consensus. Further arguments will be arbitrated by the third reviewer (ZQH). Details of the selection process will be shown in Figure 1.

#### 2.5.2 Data extraction and management

Two reviewers (ZZH and JHL) will independently extract the data from the studies. The following information will be extracted and documented in the Data Extraction Form in Excel file: basic information of the study (first author, publication year, study country, etc); study characteristics (study design, sample size, number of groups, methodology of randomization and allocation concealment, blinding, etc); participants (demographic characteristics, diagnosis, etc); interventions and controls (types of interventions, controls, duration of observation period, etc); acupuncture expectation measurement (measurement tools, type of expectation variables); treatment outcomes (definition of outcome, time point of assessment, etc); statistical methods of expectation and outcomes, and results (statistic description of acupuncture treatment, the relationship between acupuncture expectancy and outcomes, etc). If there is any disagreement during data extracting, a third reviewer (ZQH) will be required.

#### 2.5.3 Methodological quality measurement

The methodological quality measurement of each original study will be evaluated independently by 2 reviewers (JHL and ZY) by using the Cochrane Collaboration's tool for assessing risk of bias (Cochrane Manual V.5.1.0).^[23]^ The following aspects will be assessed: randomization allocation, concealment, blinding, data integrity, selective reporting, and other bias (such as trial design, baseline similarity of groups, early cessation or treatment, etc). The assessment results will be divided into 3 levels: low risk, high risk, and uncertain risk. Any discrepancy will be resolved by consensus or judged by a third reviewer (ZQH). Regarding the characteristics of acupuncture clinical trials, we will also assess the quality of acupuncture interventions according to the Standards for Reporting Interventions in Controlled Trials of Acupuncture (STRICTA) recommendation.^[24]^

#### 2.5.4 Measures of treatment effect

Suitable outcomes for different medical condition will be extracted. For dichotomous data, we will express the results for each study as the risk ratios (RRs) with 95% confidence interval (CI) and the difference or standard mean difference (SWD) with 95% CI will be applied for the continuous data.

#### 2.5.5 Dealing with missing data

Missing data will be detected and requested form the investigator of the original study. If the missing data is available, we will analyze the existing data and discuss the potential impact of missing data.

### 2.6 Statistical methods

#### 2.6.1 Assessment of heterogeneity

The heterogeneity will be evaluated by using Higgins *I*^2^ value and Chi-Square test (α = 0.1) in eligible studies. When *I*^2^ value < 50%, the heterogeneity is considered acceptable. While when *I*^2^ value > 50%, a significant heterogeneity among the included studies is considered. Because this study will include studies on acupuncture expectancy in different medical conditions, we speculate that the heterogeneity will be so great across the studies that meta-analysis could not be possibly conducted. The sources of heterogeneity that we speculate are the differences in diseases, patients’ demographics, styles of acupuncture treatment, controls, outcomes assessments, and most of all, the different assessment in acupuncture expectation and statistical methods between expectation and acupuncture response.

#### 2.6.2 Assessment of reporting biases

We will use the contour-enhanced funnel plot to assess the risk of publication bias within each pairwise comparison. If more than 10 studies are included, we will use funnel plots to assess reporting bias. If the funnel plot is asymmetric, Egger regression test will be used.^[25]^ This method will be performed in each subgroup analysis.

#### 2.6.3 Data synthesis

Considering the heterogeneity in different diseases and study design, we will conduct a descriptive systematic review rather than a meta-analysis for the eligible studies according to the result of previous studies. Statistical analyses will be conducted using the Cochrane Collaboration's software Review Manager (RevMan) V.5.3 to present direct and indirect comparisons between acupuncture treatment and controls. We will use random-effects model for data synthesis because heterogeneity will result from the diversity of diseases, interventions, study design, outcomes, and so on. If heterogeneity is significant, we will perform subgroup or sensitivity analysis for possible meta-analysis. Qualitative analysis will be conducted if there is still a big heterogeneity in subgroup analysis. In qualitative analysis, we will count the number of RCTs that reported better outcome improves connected with higher level of expectation and those did not.

#### 2.6.4 Sensitivity analysis

Sensitivity analysis will be used to test the impact of studies with high risk of bias to evaluate the robustness of pooled outcome results.

#### 2.6.5 Subgroup analysis

We plan to perform the following subgroup analyses to answer whether expectation can influence acupuncture outcomes in studies with homogeneity:

(1) Different medical conditions: We will classify different conditions for possible meta-analyses, such as pain diseases (e.g., musculoskeletal, visceral pain diseases, such as knee osteoarthritis or angina), functional disorders (e.g., functional dyspepsia, irritable bowel syndrome), psychological problems (e.g., depression), and other conditions (e.g., hot flashes, insomnia).
(2) Different types of controls: Previous studies showed that verum acupuncture has no differences to sham acupuncture, but superior to no acupuncture treatment or usual care alone. Therefore, we will classify the controls as sham acupuncture, positive medication, no acupuncture treatment/usual care, and compare with verum acupuncture to estimate whether there are more placebo effects could by acquired by sham methods in participants with higher level of expectation.
(3) Different time points of expectation measurement: In this review we will also extract data on expectation information after the last session of acupuncture treatment to evaluate whether better long-term outcomes after acupuncture treatment are associated with higher level of expectation.
(4) Different statistical methods: We will extract the different statistical methods about the acupuncture expectations. We will separately synthesis expectation data collected and analyzed in categorical and continuous variables to explore the possible different results between expectation and outcomes.

### 2.7 Ethics and dissemination

No ethical approval and patient consent are required, because all analyses were based on previous published studies. The findings of this review will be disseminated through peer-reviewed publications and conference presentations.

## 3 Discussion

With the development of complementary alternative medicine, acupuncture has been widely used in clinical practice. The specific effects of acupuncture have been controversial since some previous studies reported that there were no different therapeutic effects between verum and sham acupuncture. As 1 part of placebo effects, patient's expectation may benefit acupuncture outcomes which intrigues researchers’ interests. Although 2 systematic reviews explored the relationship between patient's expectation and outcomes in 2012^[9]^ and 2015,^[10]^ respectively, there was no confirmative conclusion due to heterogeneity. And this review needs to be updated after several new relevant studies have been published. Additionally, based on the findings of 2 previous reviews, subgroup analyses are needed for possible data synthesis based on different statistical approach, different quality of included studies, different medical conditions. For example, there are more studies on acupuncture anesthesia, in most of which more treatment benefits observed in participants with more expectations.^[6,7,11]^ We will explore whether the relationship is more significant between expectation and acupuncture responses in pain symptoms. Furthermore, we will detect whether there are different effects on outcomes when there were different expectation levels before and after acupuncture treatment. According to our previous study, patients reporting a smaller number of days with migraine attack after acupuncture treatment had a higher level of expectation. Those patients also acquired better long-term outcome improvement during the follow-up period.^[22]^

There are also some limitations in this systematic review. Firstly, we only included studies published in peer-reviewed journals in English and Chinese, which may lead to some risk of bias. Secondly, the heterogeneity may come from several aspects, such as different medical conditions, acupuncture methods, measurements. We will perform sensitivity and subgroup analyses to increase the stability of our results. Finally, considering the quality of RCTs, we only include studies published in peer-reviewed journals, which may result in higher risk of bias. We hope our findings in this review could provide more and convincing evidence in this research field.

## Author contributions

**Conceptualization:** Zuoqin Yang, Yan Li, Qianhua Zheng.

**Data curation:** Zihao Zou, Ying Zhao, Huiling Jiang, Yujun Hou.

**Formal analysis:** Yan Li, Wei Zhang, Qianhua Zheng.

**Funding acquisition:** Qianhua Zheng.

**Methodology and project administration:** Qianhua Zheng, Zuoqin Yang, Zihao Zou.

**Methodology:** Zuoqin Yang, Zihao Zou, Qianhua Zheng.

**Project administration:** Zihao Zou, Qianhua Zheng.

**Supervision:** Qianhua Zheng, Ying Li.

**Writing – original draft:** Zuoqin Yang, Yan Li.

**Writing – review & editing:** Ying Li, Qianhua Zheng.
